# A traditional Chinese patent medicine—*Suhuang zhike* capsule for cough variant asthma in adults: A protocol of systematic review and meta-analysis of randomized controlled trials

**DOI:** 10.1097/MD.0000000000018335

**Published:** 2019-12-16

**Authors:** Jianxin Wang, Rui Sun, Ruiyin Wang, Jing Han, Shuhua Zhang, Zetao Yin, Zelu Han, Ying Nong, Jiangtao Lin

**Affiliations:** a Beijing University of Chinese Medicine; b Department of Pulmonary and Critical Care Medicine, Center of Respiratory Medicine, China-Japan Friendship Hospital, National Clinical Research Center for Respiratory Diseases, Beijing, China.

**Keywords:** cough variant asthma, meta-analysis, protocol, *Suhuang* anti-tussive capsule

## Abstract

**Background:**

Many people with cough variant asthma use Traditional Chinese Patent Medicine-Suhuang anti-tussive capsule to help reduce symptoms. However there is no systematic reviews had promising its efficacy and safety for cough variant asthma.

**Methods:**

Four English databases (PubMed, Web of science, EMBASE, and Springer Cochrane Library) and 4 Chinese databases (Wanfang Database, Chinese Scientific Journal Database, China National Knowledge Infrastructure Database, and Chinese Biomedical Literature Database) were researched for the randomized controlled trials of *Suhuang* anti-tussive capsule for cough variant asthma. The search was limited to human studies, using the search keywords or free-text terms “cough,” “cough variant asthma,” “*Suhuang Zhike capsul*,”“*Suhuang anti-tussive capsul*,” and “randomized clinical trials.” Two reviewers individually extracted data from the included randomized controlled trials (RCTs). Data will be synthesized by either the fixed-effects or random-effects model according to a heterogeneity test. The primary outcomes include the frequency of asthma exacerbations during follow-up, asthmatic symptoms by validated instruments (including symptom scores, Likert scale, visual analogue scale). Lung function, serum immunoglobulin E, blood eosinophil count, phlegm eosinophil count, tumor necrosis factor-a, interleukin-1b, and adverse effects (numbers of participants experiencing each adverse events) will be assessed as the secondary outcome. Meta-analysis will be performed using RevMan5.3.5 software provided by the Cochrane Collaboration.

**Results:**

This study will provide high-quality synthesis based on current evidence of *Suhuang* anti-tussive capsule treatment for cough variant asthma.

**Conclusion:**

This analysis will provide updated evidence for whether *Suhuang* anti-tussive capsule is an effective and safe intervention for cough variant asthma.

**PROSPERO registration number:**

PROSPERO CRD42019139695.

## 1 Introduction

Chronic cough is one of the most common complains for patients seeking medical attention in both general practice and respiratory specialist clinics which affecting perhaps 5% to 10% of the adult population.^[1]^ Cough variant asthma (CVA), a phenotype of asthma and originally described as those patients with asthma and cough as the sole symptom, is the most common cause of chronic cough, which account for a higher proportion of patients with chronic cough in China than in Western countries with 32.6%,^[2]^ 44% in Japan.^[3]^

Though it shares some clinical and pathophysiological features with classic asthma with wheezing such as seasonal or nocturnal coughing, airway hyperresponsiveness, eosinophilic airway inflammation, and airway remodeling, CVA patients are more depressed and anxious than classic asthma patients.^[4]^ The most common reasons include concern about a serious underlying illness, vomiting, exhaustion, sleep disruption, social embarrassment, difficulty speaking on the telephone, urinary incontinence, and annoyance to family, friends, and workmates.^[5]^

The original definition of CVA demonstrated improvement of coughing in a small number of asthmatic subjects with bronchodilator therapy and the current GINA 2019^[6]^ and the European Respiratory Society (ERS) guidelines on the diagnosis and treatment of chronic cough in adults and children^[7]^ also recommend a short-time use of low dose inhaled corticosteroids (ICS)-formoterol or low dose ICS, and progression of CVA to classic asthma could be prevented with an early introduction of inhaled corticosteroids.^[8]^ But whether the ICS could produce a significant improvement still requires further evaluation.^[7]^ In addition, the continue treatment with ICS may cause hoarseness, pharyngeal discomfort, and candida infection and lead to the poor compliance.^[9]^ It is reported that only 52.4% of patients with CVA regularly inhaled ICS/Long-acting β2 receptor agonists within the past 6 months.^[10]^ This can lead to under-treatment of any underlying inflammation and increased risk of exacerbation. The coughing may return or be aggravated and lung function may deteriorate once the anti-inflammatory treatment is stopped. Significant benefits of oral leukotrienes receptor antagonist (LTRA) in CVA still requires further evaluation.^[11]^ An alternative antitussive strategy is to reduce hypersensitivity by neuromodulation. Low dose morphine is highly effective in a subset of patients with cough resistant to other treatments. Gabapentin and pregabalin are also advocated but in clinical experience they are limited by adverse events.^[7]^

Perhaps the most promising future developments in pharmacotherapy are drugs which shown to attenuate airway hyperresponsiveness, inflammation, bronchorelaxation, and mucus accumulation and remodeling through the use of complementary or alternative medicine (CAM). CVA patients who got poorly controlled may benefit from additional treatment options such as Chinese Herbal Medicine or Chinese Patent Medicine.

*Suhuang* antitussive capsule, also named *Suhuang zhike* capsule, which derived from Master of Traditional Chinese Medicine, Professor Chao Enxiang prescription, is composed of 9 traditional Chinese medicines including Ephedrae Herba (Mahuang), Perillae Folium (Zisuye), Pheretima (Dilong), Cicadae Periostracum (Chantui), Arctii Fructus (Niubangzi), Schisandrae Chinensis Fructus (Wuweizi), Peucedani Radix (Qianhu), Eriobotryae Folium (Pipaye), and Perillae Fructus (Zisuzi).^[12]^ It has been approved by China Food and Drug Administration (CFDA) in 2008^[13]^ and was recommended as the only one Chinese Patent Medicine to treat post-cold cough and CVA in Chinese Thoracic Society 2015 Guidelines for the Diagnosis and Treatment of Cough.^[14]^ The evidence recommended type was consistent with western medicine and *Suhuang* antitussive capsule was the first-line treatment medication in the guideline.

Though *Suhuang* antitussive capsule remain popular for CVA and its significant clinical efficacy and emerging larger market, there is no systematic reviews had promising its effects for CVA. An update of the current evidence was needed. Therefore we have conducted a Cochrane systematic review. The aim of this review is to provide an up-to-date analysis of evidence for use of *Suhuang* anti-tussive capsule for CVA in adults.

## 2 Methods

This review was prepared according to the Preferred Reporting Items for Systematic Reviews and Meta-Analyses (PRISMA) guidelines.^[15]^

### 2.1 Protocol and registration

The protocol registration number was CRD42019139695.

### 2.2 Types of studies

Studies were randomized controlled trials (RCTs) using *Suhuang* anti-tussive capsule for CVA in adult patients regardless of blinding, publication type, or language. Quasi-randomized trials, non-RCTs, cohort studies, reviews, case reports, experimental studies, expert experience were excluded.

### 2.3 Types of participants

Participants were adults with a cough variant asthma diagnosed according to the global initiative for asthma (GINA) or equivalent, such as the Experts Consensus of Chinese Medicine Diagnosis and Treatment of Asthma published by the Chinese Medical Association regardless of sex, etiology, ethnic, and course of the disease. Children with cough variant asthma were excluded.

### 2.4 Types of interventions

Interventions included *Suhuang* anti-tussive capsule alone or in combination with conventional therapy (CT) and/or biological agents for at least 2 weeks. CT was defined that prevention and treatment of Cough Variant Asthma is based on the Global Strategy for Asthma Management and Prevention (GINA) guidelines or its predecessor documents or Treatment of Asthma published by the Chinese Medical Association which includes ICS, bronchodilators, or their combinations and LTRA. The controls included no treatment, placebo, and CT alone. In addition, cointervention(s) were also included as long as they were applied in both arms.

### 2.5 Types of outcomes

RCTs had to assess at least one of the following pre-defined outcomes.

#### 2.5.1 Primary outcomes

- Frequency of asthma exacerbations during follow-up.
- Asthmatic symptoms by validated instruments (including symptom scores, Likert scale, visual analogue scale).

#### 2.5.2 Secondary outcomes

- Lung function (peak expiratory flow [PEF], forced expiratory volume in 1 second [FEV_1_], or predicted forced expiratory volume in 1 second (FEV 1%), FEV_1_/FVC [forced vital capacity]) or peak expiratory flow rate [PEFR]).
- Serum immunoglobulin E (Ig E).
- Blood eosinophil count.
- Phlegm eosinophil count.
- Tumor necrosis factor-a (TNF-a).
- Interleukin-1b (IL-1b).
- Adverse effects (numbers of participants experiencing each adverse events).

### 2.6 Search methods for identification of studies

English and Chinese databases were searched for reports of RCTs from their inception to June 2019:

- The Cochrane Central Register of Controlled Trials (CENTRAL)
- PubMed (1966 to August 2019)
- Web of science (1986 to August 2019)
- EMBASE (1980 to August 2019)
- Chinese Biomedical Database (1975 to August 2019) (http://www.sinomed.ac.cn)
- China National Knowledge Infrastructure (CNKI) (1979 to August 2019) (http://www.cnki.net/)
- VIP database (1979 to August 2019) (http://www.cqvip.com/)
- Wan fang Database (1980- August 2019) (http://www.wanfangdata.com.cn)

Reference lists of published reviews and clinical trial registries (clinicaltrials.gov, EU Registry, Chinese Registry, Australian New Zealand Registry) were also searched.

### 2.7 Searching strategy

The search strategy for PubMed was listed, which was including all search terms, and other searches will be conducted based on these results. This search strategy will be modified as required for other electronic databases.

**#1 s**earch “Cough Variant Asthma” [Title/Abstract]

**#2** Search “*Suhuang* anti-tussive” OR “*Suhuang zhike*” [Title/Abstract]

**#3** Search “RCT” [Title/Abstract] OR “randomized controlled trial” [Title/Abstract]

**#4** Search “Efficacy” [Title/Abstract] OR “safety” [Title/Abstract]

**#5** #1 and #2 and #3 and #4

### 2.8 Study selection and data collection process

#### 2.8.1 Selection of studies

**Figure 1:**
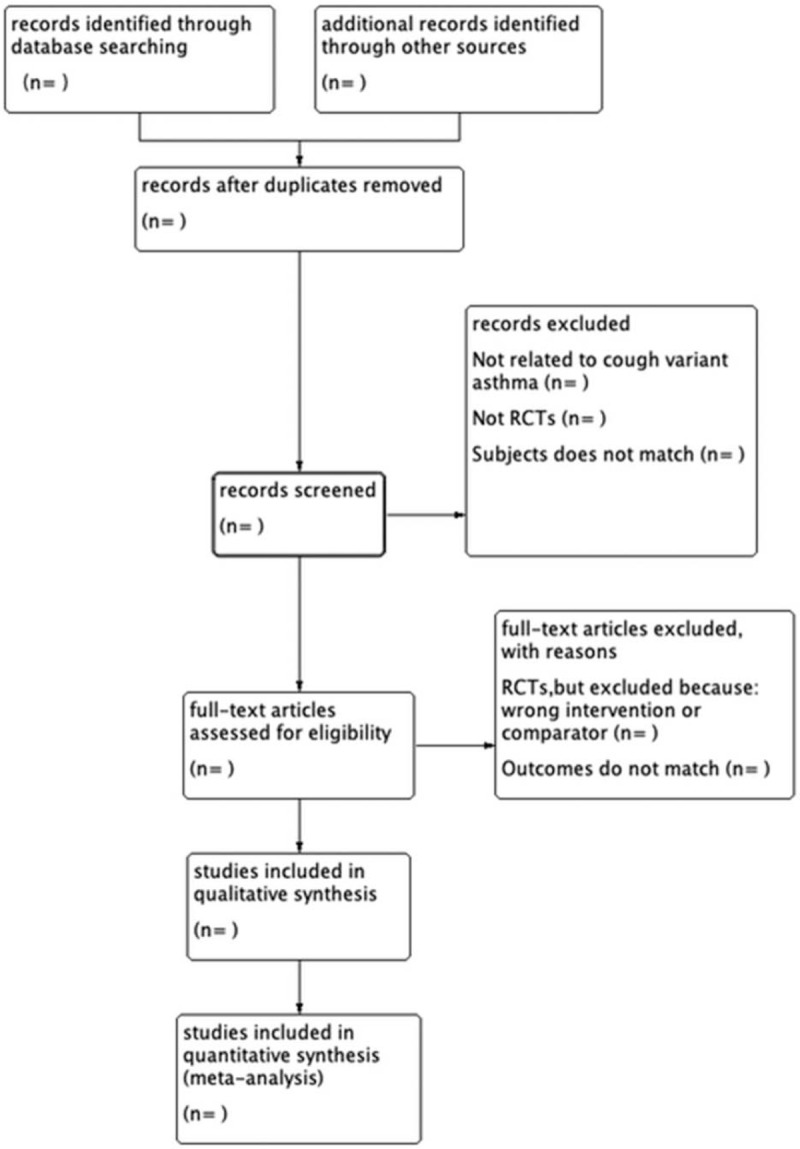
The PRISMA flow chart of the selection process. PRISMA = preferred reporting items for systematic reviews and meta-analysis protocol.

At the first stage potentially relevant trials were identified on the basis of their title, abstract, and keywords. The full-text was then obtained and assessed for inclusion or exclusion in the review. The records in this spreadsheet will be used to generate the Preferred Reporting Items for Systematic Reviews and Meta-Analyses flowchart (Fig. 1). All the procedures will be carried out by 2 independent reviewers and completed cross-check. If there is any disagreement, the 3rd author will be invited to assist in the discussion and make a decision.

#### 2.8.2 Data extraction and management

The basic process of the included literatures will be determined by referring to the Cochrane Collaboration System Evaluator's Manual (5.1.0).

Extracted data included participants (age, sex), interventions (*Suhuang* anti-tussive Capsule alone or in combination with CT), controls (type, frequency, and duration), outcomes (measures and time points), and study design (randomization, allocation concealment, blinding, and etc). If required information was not reported, we tried to request it from the corresponding author of the studies.

#### 2.8.3 Assessment of methodological quality of included studies

Two review authors independently assessed included studies for quality using 2 methods:

##### 2.8.3.1 Cochrane collaborative network system

A rigorous quality assessment of the literatures included in the study will be conducted using the bias risk assessment tool (RevMan5.3.5, the Nordic Cochrane Centre, Copenhagen, Denmark) of the Cochrane Collaborative Network System, and high, low, unclear evaluations will be performed for each item.

##### 2.8.3.2 The Jadad domains

Each trial was assessed on the following criteria:

(1) Was the study described as randomized?
(2) Was the study described as double-blind?
(3) Was there a description of withdrawal and dropouts?
(4) Was the method of randomization well described and appropriate?
(5) Was the method of double blinding well described and appropriate?

When 2 quality assessors are unable to reach a consensus on the risk assessment by negotiation, the third reviewer will make a decision.

### 2.9 Data analysis

Statistical analyses were performed using RevMan 5.3 software.

Dichotomous data. The relative risk (RR) was calculated with 95% confidence intervals (CI).

Continuous data. A fixed-effect mean difference (MD) with 95% CI was calculated for outcomes reported in the same scale, and the standardized mean difference (SMD) with 95% CI was calculated for outcomes reported in difference scales.

Assessment of heterogeneity. For the meta-analysis of non-significant heterogeneity, we applied a fixed-effect model (FEM), otherwise, Statistical heterogeneity was calculated using the *I*^2^ statistic and >50% was considered to be substantial. Subgroup analyses were performed to explore heterogeneity and a random-effects model applied.

Subgroup analysis. When heterogeneity is detected, we will judge the source of heterogeneity through subgroup analysis (e.g., different types of Chinese medicines therapies, research quality, publication age, participation population, length of treatment). In addition, we can also observe the relationship between effect values and grouping variables.

Sensitivity analysis. A sensitivity analysis was planned if sufficient studies of an intervention were identified, to examine the effect of trial quality and any quasi-randomized trials (such as alternative allocation or participants allocated on the toss of a coin), as opposed to true randomized trials (where the randomization process has been adequately concealed from the study investigators and participants).

### 2.10 Risk of bias in individual studies and publication bias

The Cochrane Collaboration's Risk of Bias Tool was used to assess bias.^[16]^ Seven domains of risk were assessed: sequence generation, allocation concealment, blinding of participants and personnel, blinding of outcome assessors, incomplete outcome data, selective reporting and other bias (baseline balance and funding/conflict of interest). Risk was assessed as low, high, or unclear by 2 independent reviewers.

Study selection, data extraction, and risk bias assessment were conducted by 2 authors independently; in case of discrepancy consensus were reached by discussion with a third party. Publication bias for meta-analysis of ≥10 studies was assessed using funnel plots and Egger test.

### 2.11 GRADE summary of findings

To summarize the quality and strength of evidence the Cochrane Grading of Recommendations Assessment, Development and Evaluation (GRADE) approach was used.^[17]^ Quality of evidence was evaluated against 5 factors: limitations in study design, inconsistency of results, indirectness of evidence, imprecision, and publication bias.

## 3 Discussion

CVA is one of the most common causes of chronic cough, and irregular treatment can lead to the progression to classic asthma. Based on the theory of “treatment according to different syndromes,” Traditional Chinese medicine has a long history and prominent characteristics and advantages in the treatment of chronic cough.

It has a relatively satisfactory clinical effect in improving clinical symptoms, signs, control of recurrence, and prevention of complications. Chinese medicine treatment as a supplementary and alternative treatment of CVA can play a role in synergizing and attenuating toxic and side effects. Compared with the decoction of traditional Chinese medicine, Chinese patent medicine which use the fixed prescription could resolve problems including the over indication, overdose, irrational combination, and incompatibility contraindications of herbs.

However, due to the complex composition of Chinese medicines, potential safety hazards may exist. The purpose of this systematic review is to systematically summarize and evaluate a large number of evidences for *Suhuang* anti-tussive capsule interventions for CVA. Evaluate the efficacy and safety of *Suhuang* anti-tussive capsule in the treatment of CVA and inform a decision aid for the clinical encounter between patients and clinicians. In addition, it may help to establish a future research agenda.

## Author contributions

**Data curation**: Rui Sun, Ruiyin Wang, Zetao Yin, Jing Han, Shuhua Zhang

**Project administration**: Jianxin Wang, Zelu Han

**Supervision**: Ying Nong, Jiangtao Lin

**Writing – original draft**: Rui Sun, Jianxin Wang

**Writing – review and editing**: Jiangtao Lin
